# Diagnosis of early neurobrucellosis using metagenomic next-generation sequencing of the cerebrospinal fluid in nonepidemic zone: Case report and lecture review

**DOI:** 10.1097/MD.0000000000041481

**Published:** 2025-02-14

**Authors:** Haijun Liu, Qianhua Li, Xia Ouyang, Qingfeng Li, Yu Min, Lie Dai

**Affiliations:** a Department of Rheumatology, Rehabilitation Medicine Institute of Panyu District The Affiliated Panyu Central Hospital of Guangzhou Medical University, Guangzhou, China; b Department of Rheumatology, Sun Yat-Sen Memorial Hospital, Sun Yat-Sen University, Guangzhou, China; c Department of Rehabilitation Medicine, Rehabilitation Medicine Institute of Panyu District The Affiliated Panyu Central Hospital of Guangzhou Medical University, China.

**Keywords:** cerebrospinal fluid, metagenomic next-generation sequencing, neurobrucellosis

## Abstract

**Rationale:**

*Brucella* neuropathy is a rare clinical condition, particularly in nonendemic areas, where it often presents with nonspecific symptoms such as fever and headaches, leading to frequent misdiagnoses. In these regions, *Brucella* antibodies are not routinely tested, and the positive rate of blood cultures is relatively low during the early stage of the disease. In addition, the low culture-positive rate of cerebrospinal fluid (CSF) means that many neurobrucellosis diagnoses rely on peripheral blood cultures or *Brucella* antibodies, which is not rigorous. This is problematic because the treatment of central nervous system brucellosis differs significantly from that of other types of brucellosis. The purpose of this study is to provide a method for the early diagnosis of *Brucella* neuropathy in nonendemic areas. We present a case of early-stage neurobrucellosis diagnosed using metagenomic next-generation sequencing (mNGS) of CSF. This approach helps avoid delaying diagnosis in the early stage in nonepidemic areas, potentially reducing the duration before diagnosis, which is of great significance for timely and appropriate treatment.

**Patient concerns:**

The patient had fever, mild headache, and a slight increase in CSF leukocytes.

**Diagnoses:**

CSF mNGS detected *Brucella*, which was later confirmed by serum *Brucella* antibody testing.

**Interventions:**

The patient was treated with rifampicin, doxycycline, and ceftriaxone.

**Outcomes:**

The patient experienced significant relief from the headache, and the fever did not recur. Subsequent examinations revealed no abnormalities in the CSF leukocytes or mNGS results.

**Lessons:**

We reviewed 10 articles on brucellosis diagnosed using mNGS, including 7 articles on neurobrucellosis (10 cases). Our review highlights the sensitivity of mNGS as a powerful tool for early detection of neurobrucellosis in the CSF. The common symptoms include fever and headache, with brain magnetic resonance imaging detecting lesions in most cases. CSF mNGS results varied, with only a few positive *Brucella* cultures or antibody tests.

## 1. Introduction

Brucellosis is the most common zoonotic infection worldwide,^[1]^ and neurobrucellosis is a rare but serious complication of the disease.^[2]^ Due to its nonspecific symptoms, traditional culture and serological tests have variable sensitivities and specificities, which can lead to misdiagnosis or delayed detection.^[2]^ The absence of distinctive clinical signs often results in the misdiagnosis of other infections, further contributing to the delayed recognition of neurobrucellosis.^[3]^

Metagenomic next-generation sequencing (mNGS) is an emerging diagnostic tool that has proven to be increasingly valuable for identifying central nervous system (CNS) infections.^[4]^ This method is particularly effective for detecting specific pathogenic bacterial infections. In this case report, we describe a patient with a brief history of fever and headache, in whom mNGS successfully detected *Brucella* in the cerebrospinal fluid (CSF). This early application of mNGS helped prevent delays in diagnosis in a nonendemic area. In addition, we reviewed several studies that used mNGS for diagnosing brucellosis, highlighting its growing role in diagnosing neurobrucellosis.

## 2. Case presentation

A previously healthy 41-year-old woman presented to our hospital on April 13, 2022, with a chief complaint of “fever and headache persisting for 26 days.” Initially, her fever peaked at 40°C but normalized after receiving antipyretic medication. However, she continued to experience recurrent fever episodes, with temperatures rising to 40°C and gradually declining after oral antipyretics. The fever was accompanied by a persistent mild headache localized to the forehead. In addition, the patient intermittently developed a rash on her left upper extremity, which often coincided with the fever. There were no signs of oral or perineal ulcers, joint pain, hair loss, or photosensitivity.

Diagnostic tests revealed an erythrocyte sedimentation rate (ESR) of 47 mm/h, C-reactive protein (CRP) of 11.56 mg/L, and hemoglobin concentration of 99 g/L. Tuberculin purified protein derivative tests at 48 and 72 hours were negative. Chest computed tomography showed ground-glass nodules in the left upper lobe and multiple small mediastinal lymph nodes.

The patient was initially treated with norfloxacin for suspected infectious fever, with body temperature normalizing. Antinuclear antibody (ANA) testing was positive (2.067; normal < 1.0), leading to referral to the Rheumatology Department for further evaluation. The patient had no abnormal family or psychosocial history.

Upon physical examination, the patient’s body temperature was 36.8°C and blood (BLD) pressure was 113/67 mm Hg. A faint red rash was noted on the left upper extremity, but no cardiopulmonary or abdominal abnormalities were found. Muscle tone and strength were normal, and no signs of meningeal irritation were observed, despite positive bilateral Papanicolaou signs.

BLD tests showed a white blood cell (WBC) count of 3.59 × 10^9^/L and hemoglobin of 99 g/L. Urine analysis indicated PRO and BLD+++. Routine stool tests were normal, and liver and kidney functions were within the normal range. CRP, ESR, and procalcitonin levels were 23.3 mg/L, 50 mm/h, and 0.19 ng/mL, respectively. Screening for antibodies and nucleic acids of common pathogens, including cytomegalovirus, Epstein–Barr virus, and others, was negative. Urine culture was negative, and immunofluorescence testing for ANA, extractable nuclear antigens, and antineutrophil cytoplasmic antibodies was also negative.

The patient, a previously healthy individual, presented with fever and mild headache, without symptoms affecting other organs. Given these signs and suspicious pathological findings, we initially suspected a CNS infection. A lumbar puncture was performed, revealing normal intracranial pressure (160 mmH_2_O). The CSF was colorless and transparent, with a WBC count of 14 × 10^6^/L, 21% mononuclear cells, and 79% segmented neutrophils. Pandy test was negative, and no *Cryptococcus* infection was detected. CSF biochemistry revealed adenosine deaminase, chloride, glucose, and lactate dehydrogenase levels of 0.2 U/L, 125.1 mmol/L, 2.96 mmol/L, and 13 U/L, respectively. Enhanced cranial magnetic resonance imaging (MRI) and magnetic resonance angiography scans were unremarkable. However, the elevated WBC count suggested viral meningitis, and we started acyclovir treatment while awaiting further diagnostics.

On the third day of admission, mNGS of the CSF identified *Brucella abortus* (2/19353). Given the limited number of sequences, serum brucellosis antibody testing was conducted. By the 5th day, the Rose Bengal plate test was positive, and the tube agglutination test showed a titer of 1:400 with positive IgG and IgM antibodies. Despite negative CSF and BLD cultures, neurobrucellosis was confirmed.

Epidemiologically, the patient had significant exposure in the 2 weeks before symptom onset. She had cleaned a turtle pond in her yard, during which sewage accidentally splashed into her eyes. In addition, she had applied flower fertilizer containing sheep manure to yard flowers 3 months earlier. Rainwater from the flowerpots may have flowed into the turtle pond. Although she denied direct contact with cattle, sheep, or consumption of fresh mutton, her family members tested negative for brucellosis antibodies. Given the circumstances, it is likely that the pathogenic bacteria were transported from the flowerpot to the turtle pond by rainwater, and the patient became infected while cleaning the pond.

Treatment was initiated with a combination of doxycycline (100 mg twice daily), rifampicin (600 mg daily), and ceftriaxone (2 g twice daily) after mNGS identified *Brucella* in the CSF. After 3 days of treatment, routine BLD tests showed a WBC count of 2.87 × 10^9^/L, absolute neutrophil count of 0.88 × 10^9^/L, and hemoglobin level of 81 g/L. Bone marrow cytological analysis revealed normal hyperplasia with few myeloid cells and no megakaryocytes. Bone marrow culture was normal. After 1 week of treatment, repeat CSF analysis (including routine, biochemical, culture, and mNGS tests) showed no abnormalities and a leukocyte-raising drug was administered to normalize leukocyte levels.

The patient received ceftriaxone for 6 weeks, and rifampicin and doxycycline for 6 months. A 1-year follow-up showed no recurrence of fever, headache, hearing loss, or blurred vision. BLD tests revealed normal WBC count, ESR, and CRP levels. Repeated ANA testing (via enzyme-linked immunosorbent assay and immunofluorescence) was negative. In addition, head MRI re-examination, including enhanced scanning, showed normal findings. The treatment timeline is summarized in Table 1.

**Table 1:** The timeline of episode and treatment of the patient.

Time	Clinical manifestations	Tests result	Treatment
March 18, 2022	Started fever with mild headache	CRP and ESR was normal	Antipyretic medication, had no fever for several days, still had mild headache
April 2, 2022	Fever, had mild headache	ESR: 47 mm/h, CRP: 11.56 mg/h, PPD: 48 and 72 hours (−), no obvious abnormalities in chest CT	Ninofloxacin, had no fever for several days, still had mild headache
April 11, 2022	Fever, mild headache	ANA 2.067 (+, ELISA)	
April 13, 2022	Fever, mild headache		Admitted
April 14, 2022	Fever, mild headache	CRP: 23.3 mg/h, WBC of CSF: 14 × 10^6^/L, ANA: (−, IIF), cranial MRI is normal, CSF was sent for mNGS	Acyclovir
April 15, 2022	No fever, mild headache	mNGS of CSF identified *Brucella abortus*	Rifampicin (600 mg daily), doxycycline (100 mg twice daily), ceftriaxone (2 g twice daily) were started
April 17, 2022	No fever and headache	Brucellosis antibody tests were positive	
April 19, 2022		Blood culture was negative	
April 23, 2022		Blood WBC was 2.87 × 10^9^/L, no obvious abnormality in bone marrow cytological analysis, bone marrow cultures was normal	Added leukocyte-raising drug
April 28, 2022		Blood culture, CSF culture and mNGS of CSF all negative, CRP: 1.4 mg/L, ESR: 32 mm/h	Discharged
May 30, 2022		Blood WBC, CRP, ESR were normal	Stopped ceftriaxone, rifampicin, and doxycycline continued
October 20, 2022		Blood WBC, CRP, ESR were normal, cranial MRI was normal	Stopped rifampicin and doxycycline
April 11, 2023		Blood WBC, CRP, ESR were normal, cranial MRI was normal, ANA (IIF) was negative	

ANA = antinuclear antibody, CRP = C-reactive protein, CSF = cerebrospinal fluid, ELISA = enzyme-linked immunosorbent assay, ESR = erythrocyte sedimentation rate, IIF = indirect immunofluorescence assay, mNGS = metagenomic next-generation sequencing, MRI = magnetic resonance imaging, PPD = tuberculin purified protein derivative test, WBC = white blood cell.

## 3. Literature review

A comprehensive search of the PubMed platform was conducted till December 20, 2024, using the search terms “(neurobrucellosis OR brucellosis OR *Brucella melitensis*) AND (metagenomic next-generation sequencing)” The search yielded 14 articles, 11 of which were relevant to brucellosis. Ten articles were categorized as case reports or collectively documented cases, including 16 cases.^[4-13]^ Furthermore, 7 of the 10 articles specifically focused on neurobrucellosis and encompassed a total of 10 cases.^[4,5,7-9,11,12]^

## 4. Results

Table 2 summarizes the characteristics of the 17 cases included in this study, including the present patient, with 7 men and 10 women. The youngest patient was 6 years old, with an average age of 40.71 ± 20.84 years. The average duration from symptom onset to brucellosis diagnosis was 5.38 ± 7.84 months, ranging from 8 days to 24 months. Among the cohort, 11 patients were diagnosed with neurological brucellosis. Fever and headache were the most common symptoms in the 17 patients with neurobrucellosis, although 2 patients presented without fever, exhibiting symptoms such as hearing loss, headache, and weight loss. Other neurological symptoms included fatigue, vomiting, blurred vision, coma, and convulsions. Musculoskeletal symptoms, including lower back and joint pain, were reported in most patients.

**Table 2:** Clinical characteristics of 17 patients with brucellosis diagnosed using metagenomic next-generation sequencing.

Author	Age, gender	Diagnoses	Time from onset to CSF collection (mo)	Main symptoms	Main laboratory results	Cranial MRI findings	Sample for mNGS	*Brucella* species-specific reads/raw reads (genomic coverage)	Therapeutic regimens	Outcome	Others
Fan et al^[4]^	38,M	Neurobrucellosis	24	Fever, headache, hearing loss, back pain	NA	CNS involvement, stroke, meningitis	CSF	30/22865302 (0.11%)	NA	NA	
	46,F	Neurobrucellosis	12	Hearing loss, weight loss	NA	CNS involvement, stoke	CSF	11/19083456 (0.043%)	NA	NA	
	38, F	Neurobrucellosis	6	Headache, hearing loss	NA	Meningitis, brain abscess	CSF	24/24691240 (0.093%)	NA	NA	
	49, M	Neurobrucellosis	4	Fever, seizures	NA	Meningitis, encephalitis	CSF	104/30738055 (0.4%)	NA	NA	
Mongkolrattanothai et al^[5]^	11,F	Neurobrucellosis	24	Fever, headache, nausea, back pain	Blood *Brucella* EIA IgG(+), CSF X-Pert (−), CSF TB culture (−)	Myelitis	CSF	277/23638587 (0.0012%)	Doxycycline and rifampin	Cure	Initial treatment was for tuberculous meningitis; however, the response was unsatisfactory
Xi et al^[6]^	57, F	Endopthalmitis of Brucella	1.5	Right-eye vision loss	*Brucella* standard tube agglutination was 1:25		Aqueous humor	13660/13762 (99.26%)	Doxycycline and rifampin	Cure	
Cao et al^[7]^	39, M	Neurobrucellosis	2	Headache, dizziness, paralysis of both the lower limbs and blurred Vision, obnubilation, and restlessness	No specific results for *Brucella*	No remarkable abnormality was found in brain MRI	CSF	199/21542240 (0.009%)	Doxycycline, sulfamethoxazole, and amoxicillin-clavulanic acid, switch to doxycycline, rifampin, and cefatriaxone later	Cure	
Geng et al^[8]^	6, M	Meningoencephalitis, conjunctivitis, coronary artery aneurysm	<1 (20 d)	Fever, fatigue, and drowsiness	T-spot negative, blood culture showed *B. melitensis*		CSF	1/124657 (0%)	Rifampicin, sulfamethoxazole and ceftriaxone	Cure	
Mathias et al^[9]^	32, M	Neurobrucellosis	1	Fever, headache, dizziness, and memory complaints	Brain biopsy showed macrophage infiltration, no obvious granuloma	Deep left nucleocapsular gliosis and acute ischemic lesions were absent	CSF	486/2191 (22.18%)	Ceftriaxone, rifampicin, doxycycline, and dexamethasone	Cure	Upon administration of ceftriaxone, rifampicin, and doxycycline, he experienced a severe headache, prompting the addition of dexamethasone
Du et al^[10]^	68,F	Brucellosis	3	Back pain	RBT (+)		Tissue	20/247467502 (0%)	Rifampicin, doxycycline	Cure	
Wu et al^[11]^	10, F	Neurobrucellosis	<1 (2 wk)	Fever, headache	CSF culture yielded *Brucella*, *Brucella* antibody testing was 1:400	Slightly elevated signals in portions of the bilateral frontal and temporal insular cortices	CSF	32/299 (10.7%)	Doxycycline, rifampin, and cefatriaxone	Cure	
Chen et al^[12]^	64, F	Neurobrucellosis	<1 (8 d)	Left limb weakness	Blood culture showed *B. melitensis*	Multiple intracranial demyelination lesions and infarction only in the right temporal lobe and posterior horn of the ventricle	CSF	NA	Doxycycline, rifampin, and cefatriaxone	Cure, but with left limb disable	
Zhang et al^[13]^	60, M	Brucellosis	<1 (10 d)	Fever	No specific results for *Brucella*		Blood	4/NA	Omadacycline, levofloxacin	Cure	
	60, F	Brucellosis	2	Fever	RBT (+) and tissue culture showed *Brucella*		Tissue	1314/NA	Doxycycline, rifampin	Cure	
	71, F	Brucellosis	1	Back pain	Abscess fluid culture showed *Brucella*		Tissue	30/NA	Doxycycline, rifampin	Cure	
	55, M	Brucellosis	3	Back pain	RBT (+)		Tissue	58/NA	doxycycline, rifampin		
The present patient	41, F	Neurobrucellosis	<1 (26 d)	Fever, mild headache	Blood *Brucella* EIA IgG(+)	No remarkable abnormality was found in brain MRI	CSF	2/19353 (0.01%)	Doxycycline, rifampin, and cefatriaxone	Cure	

CSF = cerebrospinal fluid, EIA = enzyme immunoassay, F = female, M = male, mNGS = metagenomic next-generation sequencing, MRI = magnetic resonance imaging, NA = not available, RBT = Rose Bengal test, T-spot = T cell spot test.

A total of 10 of the 11 patients of neurobrucellosis underwent cranial MRI, of whom 2 had normal findings (at 2 months and 26 days of disease duration), while the other 8 showed MRI abnormalities, such as myelitis, stroke, meningitis, abscess, cranial nerve involvement, and encephalitis. Notably, patients with meningitis who exhibit abnormal findings on MRI tent to have a longer disease course (>4 months).

mNGS was performed on specimens from all 17 patients, including intraocular fluid from 1 patient, BLD from 1 patient, tissues from 4 patients, and CSF from the remaining 11. The read counts ranged from 1 (in a patient with a 20-day disease course) to 13,660 (in a specimen taken from intraocular fluid at 1.5 months of disease). Among the 11 neurobrucellosis patients, 3 had a positive CSF culture for *Brucella*, 3 had positive BLD cultures, and 6 showed positive *Brucella* antibodies or Rose Bengal test (RBT) results (Table 3). The mNGS results from the CSF of these patients showed a minimum of 1 read, a maximum of 486, and an average of 116.60 ± 185.08.

**Table 3:** Characteristics of the cerebrospinal fluid in 11 patients with neurobrucellosis.

Author	Age, gender	CSF						Peripheral blood	
		Pressure (mmH_2_O)	WBC (cell/μL)	MN (%)	Protein (g/L)	Glucose (mmol/L)	Culture	Culture	Antibodies/RBT
Fan et al^[4]^	38, M 46, F 38, F 49, M	210 >330 >330 220	607 361 178 86	99 91 99 93	9.3 3.14 1.09 1.28	1.2 0.7 0.8 1.7	*Brucella* – – –	– – – –	−/− −/+ −/+ −/+
Mongkolrattanothai et al^[5]^	11, F	NA	183	3.7	2.7	0.25	–	NA	+/NA
Cao et al^[7]^	39, M	300	210	NA	4.39	5.29	–	–	−/NA
Geng et al^[8]^	6, M	NA	NA	NA	NA	NA	–	*B. melitensis*	−/+
Mathias et al^[9]^	32, M	NA	44	NA	0.82	2.55	–	NA	NA
Wu et al^[11]^	10, F	NA	259	4.6	0.78	1.66	*Brucella*	–	+/NA
Chen et al^[12]^	64, F	NA	NA	NA	3.74	NA	*Brucella*	*Brucella*	NA/–
The present patient	41, F	160	14	21	0.25	2.96	–	*Brucella*	+/+

CSF = cerebrospinal fluid, F = female, M = male, MN = mononuclear, NA = not available, RBT = Rose Bengal test, WBC = white blood cell.

All 17 patients were treated with rifampicin and doxycycline. In 4 patients with severe neurobrucellosis and in the present case, ceftriaxone was added, while another patient received sulfamethoxazole. Notably, all patients showed clinical improvement following treatment.

## 5. Discussion

We report a case of early-stage neurobrucellosis diagnosed by mNGS in a nonendemic area, and also review cases of neurobrucellosis diagnosed by mNGS. Our results suggest that common manifestations of neurobrucellosis include fever and headache, which may be accompanied by lower back pain and arthralgia, but atypical symptoms such as hearing loss and weight loss can also occur. Brain MRI can detect lesions in most cases, although a few cases show no abnormalities on MRI. In the 11 neurobrucellosis patients, the CSF mNGS results showed a minimum of 1 read and a maximum of 486, averaging 116.60 ± 185.08. Among these cases, only 3 had positive *Brucella* cultures in CSF, and 6 had positive *Brucella* antibodies or RBT results. All patients with neurobrucellosis had good therapeutic outcomes, but those presenting with cerebral infarction would be left with hemiplegia. Our case report highlighted the diagnostic challenges of neurobrucellosis, especially in nonendemic areas, and demonstrated the effectiveness of mNGS in early diagnosis, even in patients with short disease duration and mild symptoms. The treatment outcomes were positive, with patients’ symptoms resolving and follow-up examinations showing no abnormalities.

This report presents a case of neurobrucellosis potentially triggered by inadvertent eye exposure to contaminated water while cleaning a turtle pond, which was contaminated with sheep manure. The established diagnostic criteria for neurobrucellosis are as follows^[2]^: clinical presentation consistent with recognized neurobrucellosis syndromes; characteristic CSF changes, including mild elevation in WBC count and elevated protein levels; positive BLD, bone marrow, or CSF cultures, or positive serological tests (e.g., BLD agglutination test titer ≥1:160 or positive CSF titer); clinical improvement following appropriate treatment; and exclusion of other potential diagnoses.

In this case, the patient presented with fever and headache, with the fever displaying a typical undulating pattern. Serum antibodies against *Brucella* were detected, and the symptoms completely resolved after treatment. The patient met all the diagnostic criteria for neurobrucellosis (meningitis).

Given the patient’s presentation, it was crucial to consider alternative diagnoses. Initially, the positive ANA via enzyme-linked immunosorbent assay, combined with a rash, raised the possibility of a connective tissue disease. However, ANA testing using immunohistochemical methods was negative, making the likelihood of a connective tissue disorder minimal. In addition, the slight elevation in the WBC count in the CSF initially raised concerns about viral meningitis. However, the detection of *Brucella* in the CSF effectively ruled out viral meningitis. In this case, neurobrucellosis was diagnosed swiftly through mNGS of the CSF, despite the short disease duration and relatively mild symptoms. Diagnosing neurobrucellosis in nonendemic areas, particularly in its early stages, is challenging. Most medical literature reports neurobrucellosis cases of moderate-to-severe severity, with a median symptom duration of 1 month in endemic zones,^[14]^ and a duration of 2 months or more in nonendemic zones.^[4,5]^ Therefore, this case may represent one of the shortest duration cases diagnosed in a nonendemic area.

A few early-stage cases have been diagnosed, but these typically rely on repeated serum antibody tests or CSF culture, which often yield negative results in the early stage.^[15]^ Diagnosing neurobrucellosis in these cases often requires a combination of positive serum antibodies or BLD cultures with neurological symptoms and diagnostic signs.^[16]^ However, detecting *Brucella* directly in the CSF is more reliable than relying solely on these methods. Given the differences in treatment protocols and durations between neurobrucellosis and nonneurobrucellosis cases,^[17]^ early identification of *Brucella* in the CSF is crucial. Despite its rarity, early-stage detection of *Brucella* in the CSF remains infrequent, particularly in nonendemic areas. CSF cultures have a relatively low positive rate of ≈14%.^[3]^ In contrast, mNGS has shown much higher sensitivity. A study from the northwestern region of China, an endemic area for brucellosis, found that from 2015 to 2021, the sensitivity of mNGS in detecting *Brucella* in CSF was 90%, compared with 54.5% for CSF culture. This study had a higher culture positivity rate could be attributed to a longer disease duration before diagnosis (average of 4.5 months).^[18]^ Therefore, mNGS is highly recommended for diagnosing neurobrucellosis, especially in nonendemic regions.^[3]^

mNGS is a cutting-edge technique that is increasingly used in the clinical diagnosis of CNS infectious diseases, often yielding “unexpected” results.^[4]^ Several studies have applied mNGS for diagnosing brucellosis and neurobrucellosis. We reviewed 10 articles that focused on using mNGS for brucellosis or neurobrucellosis diagnosis. Of these, 7 specifically addressed neurobrucellosis, covering 10 cases. Notably, in 2016, Mongkolrattanothai et al^[5]^ used mNGS to correctly diagnose neurobrucellosis in a patient who had been misdiagnosed with tuberculous meningitis.

Among these cases, patients with longer disease durations had higher numbers of *Brucella* reads in the CSF through mNGS, while the patient with the shortest disease duration (10 days) had only 4 sequence read. Fever and headache are common symptoms of neurobrucellosis, but not all patients present with these symptoms, complicating the diagnosis. In addition, while cranial MRI can reveal lesions associated with neurobrucellosis, not all patients exhibit distinct infectious lesions on imaging, particularly in early-stage cases (even within a 2-month disease course), which may lead to normal results.

All 11 neurobrucellosis patients, including the present case, underwent mNGS. Among them, only 3 patients had a positive *Brucella* CSF culture, 3 had a positive *Brucella* BLD culture, and 6 showed positive *Brucella* antibodies or RBT results in peripheral BLD. After receiving a confirmed diagnosis, all patients were treated with a combination of rifampicin and doxycycline, leading to improvement. This highlights that once neurobrucellosis is accurately diagnosed, prognosis generally improves.

These cases underscore the diagnostic challenges of neurobrucellosis, where CSF or peripheral BLD cultures often yield low positivity rates. mNGS presents a promising tool for enhancing diagnostic accuracy, especially in early or atypical cases in nonendemic areas.

The treatment of neurobrucellosis typically requires combination therapy, as monotherapy often results in relapses.^[2]^ Commonly used regimens include rifampicin (600 mg daily), doxycycline (100 mg twice daily), ceftriaxone (2 g twice daily), and sulfamethoxazole (800 mg twice daily). Doxycycline is favored due to its ability to effectively cross the BLD–brain barrier and its prolonged half-life. In this case, the patient was treated with rifampicin, doxycycline, and ceftriaxone. Ceftriaxone was administered for 6 weeks, while rifampicin and doxycycline were continued for 6 months. Following the initiation of treatment, the patient’s fever subsided, and the severity of her headache gradually diminished. The observed leukopenia prompted a bone marrow cytological analysis, which revealed normal leukocyte proliferation. Leukopenia was attributed to brucellosis and justified continued anti-infection therapy. During the outpatient follow-up, the patient remained free of fever, her headaches alleviated, and her WBC count normalized. A year later, a repeat enhanced cranial MRI showed no abnormalities.

The strength of this case lies in the early diagnosis of neurobrucellosis using mNGS in a nonepidemic area, despite the absence of obvious symptoms and clear epidemiological history. However, there are several limitations to this study that should be acknowledged. First, the patient’s epidemiological history is not entirely clear. This lack of clarity may limit the generalizability of the findings and the understanding of the disease’s transmission dynamics in nonendemic settings. Second, during the literature review process, we only included case reports with detailed case information. Two series of case characteristic analyses, specifically those focusing on the role of mNGS in the diagnosis of brucellosis, were not incorporated into our analysis. The reason for excluding these series was the lack of detailed case information in them, which made it difficult to draw specific conclusions relevant to the focus of our study, and they did not include cases of neurobrucellosis. While this does not impact the conclusions of our article. These limitations should be taken into account when interpreting the scope and findings of this study, as they may influence the applicability of our results to other similar cases and the completeness of the literature synthesis provided.

In conclusion, we present a case of mild neurobrucellosis in its early stages, demonstrating the potential of mNGS to improve pathogen detection in nonendemic regions. The key takeaway is that when a CNS infection is suspected, it is essential to promptly perform mNGS on CSF, particularly in cases with a short disease duration. This approach may help increase the rate of positive pathogen detection.

## Acknowledgments

The authors thank the patient featured in this article for agreeing to share her case information to promote medical progress.

## Author contributions

**Conceptualization:** Haijun Liu, Yu Min.

**Formal analysis:** Haijun Liu, Yu Min.

**Investigation:** Haijun Liu, Qingfeng Li.

**Validation:** Haijun Liu.

**Writing – original draft:** Haijun Liu, Qianhua Li, Xia Ouyang.

**Writing – review & editing:** Haijun Liu, Qianhua Li, Lie Dai.

**Data curation:** Xia Ouyang.

**Resources:** Xia Ouyang, Qingfeng Li.

**Methodology:** Yu Min, Lie Dai.

**Project administration:** Lie Dai.

**Supervision:** Lie Dai.

**Visualization:** Lie Dai.
